# Being a dad to a child with Down’s syndrome: Overcoming the challenges to adjustment

**DOI:** 10.1111/jar.12563

**Published:** 2019-01-11

**Authors:** Anna Ridding, James Williams

**Affiliations:** ^1^ University of Liverpool Liverpool UK

**Keywords:** adjustment, Down’s syndrome, fathers, intellectual disability, paternal

## Abstract

**Background:**

Historically, research exploring the impact of having a child with an Intellectual Disability (ID), has focussed exclusively on mothers. The present study aimed to investigate fathers’ experiences of parenting a child with Down's syndrome (DS), their contributions, influences on family functioning and inclusion in their child's support provision.

**Method:**

Semi‐structured interviews were conducted with 15 fathers. Interviews were analysed using Grounded Theory (GT).

**Results:**

Fathers’ adjustment appeared to be on a fluid trajectory with three key categories influencing this: “Accommodating the Child,” “Adapting the Parental/Spousal Role” and “Adapting Society.”

**Conclusions:**

The accounts uncovered fathers’ adjustment trajectory to parenting a child with DS, concluding that despite practical and emotional challenges, fathers employed strategies to achieve positive adjustment. Fathers identified the need for services to recognize their role and involve them in their child's support provision. Implications for clinical practice and future research are discussed.

## 1 INTRODUCTION

For years, society has failed to facilitate men's parenting roles, despite changes in social circumstances and cultural values which have seen an increase in the active involvement of fathers in their children's care (Lillie, 1993; Machin, 2015; Schoppe‐Sullivan, McBride, & Ringo Ho, 2004). There remains limited research focussing exclusively on fathers as carers, with studies often relying on indirect reports from mothers (e.g., Redshaw & Henderson, 2013). The small amount of existing research focuses predominantly on fathers of children without an intellectual disability (ID) and highlights the importance of paternal involvement for children's and fathers’ development and satisfaction (Brown, Mangelsdorf, & Neff, 2012; Palm & Fagan, 2008; Pleck & Masciadrelli, 2004). Researchers claim that the lack of paternal involvement in research is due to them being seen as unworthy of researching in their own right or, alternatively, “hard to reach” (e.g., Carpenter & Towers, 2008; May & Fletcher, 2013; Ricci & Hodapp, 2003). Carpenter and Towers (2008) highlight that fathers remain stereotyped by outdated societal assumptions that their role is a protector and provider, with limited emotional involvement. Moreover, researchers have suggested that employers and services reinforce these beliefs limiting fathers’ inclusion leading to restricted accessibility for researchers (Brown et al., 2012).

There continues however to be a rise in fathers’ active involvement in childcare, with an increase in the participation of women in the workforce (Pleck & Masciadrelli, 2004). This study hopes to further highlight the availability of fathers and the need to recognize and research their important roles.

## 2 THE IMPACT OF PARENTING A CHILD WITH AN INTELLECTUAL DISABILITY

Historically, research exploring the impact of an ID on the family regarded mothers’ experiences as representing the whole family (Hastings, 2003). This research highlighted increased maternal distress often closely linked with uncertainty related to the child's diagnosis and transition points (Baker, Blacher, Crnic, & Edelbrock, 2002; Baxter, Cummins, & Polak, 1995; Gallimore, Weisner, Bernheimer, Guthrie, & Nihira, 1993). Spousal well‐being further affects parent experiences, for example research has shown that parents who are pessimistic and exhibit external locus of control report lower parental adjustment scores and more stress when caring for a child with an ID (Baker, Blacher, & Olsson, 2005; Lanfranchi & Vianello, 2012). Additionally, Cummings (1976) suggests that the child's age impacts upon parental experiences with fathers of older children with an ID exhibiting lower levels of stress and depression.

Furthermore, it is suggested that parental adjustment is achieved over time and is affected by employment and the parent's ability to make adaptations to roles (Carpenter & Towers, 2008; Foundation for People with Learning Disabilities [FLPD], 2005; West, 2000).

Much less is known specifically about father's well‐being.

## 3 FATHERS OF CHILDREN WITH INTELLECTUAL DISABILITIES

A number of studies exploring experiences of parents of children with an ID suggest that fathers’ and mothers’ experiences differ, particularly in regards to stress (Bailey, Blasco, & Simeonsson, 1992; Lanfranchi & Vianello, 2012). This could be attributable to the respective variables predicting stress. Fathers have been shown to experience more stress attributed to their child's delay in social skills and acceptability (Lanfranchi & Vianello, 2012). Alternatively, maternal paternal differences might be attributable to differences in coping strategies, such as fathers concealing their emotions to protect their family (Barak‐Levy & Atzaba‐Poria, 2013; Houser & Seligman, 1991) and dealing with their difficulties alone due to less accessible support (Parette, Meadan, & Doubet, 2010; Pelchat, Lefebvre, & Perreault, 2003).

It should be noted however that, similar to mothers, fathers of children with ID recognize their personal growth and the positive aspects of parenting (Carpenter & Towers, 2008; Hornby, 1992).

Rodrigue, Morgan, and Geffken (1992) suggest that the child's gender affects fathers’ parenting experiences; however, this has not been supported elsewhere (Houser & Seligman, 1991). Discrepant findings may be attributed to the differences of the children's ages in each of these studies. For example, research suggests that fathers’ distress levels and coping strategies change in line with children's development with fathers initially relying on the Internet to cope with difficult feelings at birth and later turning to support groups to ameliorate distress (Cummings, 1976; Harrison et al., 2007; Rendall, 1997; West, 2000). Rodrigue et al. (1992) conducted a cross‐sectional study with 20 American fathers of younger children (mean age of 11.9), using measures to assess coping, competence, marital satisfaction, support and family cohesion. Comparatively, Houser and Seligman (1991) recruited 40 fathers with children with a mean age of 15.3. It is therefore possible that they experienced different stressors due to their child's age which in turn highlighted or masked the impact of a child's gender. In contrast, a more recent study exploring 66 fathers’ experiences of parenting ID and non‐ID children over several years observed no changes in fathers’ distress as the child develops. It is possible these discrepant findings are attributable to how each study defined, measured and analysed stress. For example, both Cohen, Zeedyk, Tipton, Rodas, and Blacher (2016) and Houser and Seligman (1991) utilized a specific measure to assess psychological symptoms and stress, whereas Rodrigue et al. (1992) drew conclusions from what could be seen as inappropriate measures.

There is a dearth of awareness around the experiences of fathers in the United Kingdom (UK): existing research is limited due to being conducted overseas, and predominantly utilizes self‐report questionnaires to explore limited constructs within father's experiences, for example focusing simply on challenges and stress. This study hopes to focus on father's adjustment more broadly and provide in‐depth exploration of father's experiences about parenting a child with Down's syndrome.

## 4 FATHERS OF CHILDREN WITH DOWN'S SYNDROME

Down's syndrome (DS) is a genetic condition, resulting from an extra chromosome 21 in each of the body's cells. The speech and language of children with DS often develops slowly and most will have mild to moderate levels of ID (Dykens, Hodapp, & Finucane, 2000).

Research exploring the specific impact of parenting a child with DS is limited, due to samples focusing mainly on mothers and frequent inclusion of fathers in broader studies covering other IDs and autistic spectrum conditions (ASCs). Parenting a child with DS may evoke different responses to other IDs which studies combining different diagnostic groups fail to fully explore (Cuskelly, 1999; Hodapp & Dykens, 2012). For example, Hartley, Seltzer, Head, and Abbeduto (2012) conducted a cross‐sectional study in America comparing fathers of teenagers with Autism (*n* = 135), DS (*n* = 59) and Fragile X Syndrome (*n* = 46). They referred to a “DS advantage” whereby fathers report lower stress levels, more positive views and more support‐seeking strategies compared to fathers of children with other IDs. Researchers attribute this to the child's personality, frequent hospitalizations which provide opportunities to strengthen the parental bond, and support group availability (Derrington et al., 2013; Sullivan, 2002).

There is a dearth of literature exploring the specific factors involved in the adjustment of fathers of children with DS. Most of the studies which solely recruit this population highlight that, despite challenges particularly at the time of birth, fathers adapt positively (Bentley, Zvonkovic, McCarty, & Springer, 2015; Gault, 2009; Henn & Piccinini, 2010; Herbert & Carpenter, 1994; Hornby, 1995). Moreover, fathers’ overall adjustment appears to be associated with spousal support and satisfaction, the child's behavioural difficulties, the fathers’ employment status and his subsequent finances (Cohen et al., 2016; Hornby, 1995; Rodrigue et al., 1992).

To cope with challenges, fathers employ a range of strategies such as attending support groups, although it is recognized that father orientated support is limited (Gault, 2009; Herbert & Carpenter, 1994; Rodrigue et al., 1992).

While these studies add to the research literature, a number of these studies utilize cross‐sectional methodologies and quantitative measures which do not provide an in‐depth understanding of fathers’ experiences to the same extent as qualitative methods (Cuskelly, Hauser‐Cram, & Riper, 2008). Furthermore, as fathers’ roles in families are changing, we can hypothesize so too will their adjustment experiences and the conclusions of the research above may become outdated. The current study is therefore designed to gain a current qualitative understanding of the processes of fathers’ adjustment to parenting a child with DS.

## 5 AIMS AND RESEARCH QUESTIONS

The aim of this study is to develop a model to account for fathers’ experiences of parenting a child with DS: their contributions; influences on family functioning; and inclusion in their children's support provision. The following research questions will be addressed:

1. How do fathers adjust to living with a child with DS and what parenting roles do they play?
2. What specific factors contribute to their adjustment and how?

Current research predominantly focuses on and measures narrow constructs, for example father's stress or marital satisfaction, and this study hopes to provide a broader, more in‐depth exploration of father's adjustment to a child with DS. To the best of the author's knowledge, this is the first study to propose a theoretical model to capture father's experiences about raising a child with DS.

## 6 METHOD

This study aimed to explore paternal adjustment to parenting a child with DS. Grounded Theory (GT) was considered most congruent with the study aims and allowed the researchers to examine experiences in depth, while contributing to a theoretical understanding and development of a model (Kennedy & Lingard, 2006). The analytical methods outlined by Strauss and Corbin (1998) were utilized to guide the analysis; open, axial and selective coding. The first stage of the study involved semi‐structured interviews using broad‐ and open‐ended questions, allowing themes to develop and deepen. Simultaneous data collection and analysis allowed the constant comparison of themes both within and between participants. Open coding involved generating initial concepts and the construction of categories. Axial coding involved the development and linking of categories. Finally, selective coding required formalizing relationships between categories into a theoretical framework. Although the study followed the coding and analytical procedures outlined by Strauss and Corbin (1998), the authors acknowledge the role played by the research in the co‐construction of meaning with the participant (Charmaz, 2000).

## 7 SAMPLE

The sample consisted of fifteen fathers of children with DS, recruited from support groups within the North West of England. Participants were aged from 26 to 52 years old (*M* = 40.6 years old) and were able to communicate verbally in English. Additionally, fathers were only recruited if they had a birth child under ten years old, to allow for some variation in a recent and well‐defined cohort. It was felt that fathers with older children might be at a different stage of adjustment (Cummings, 1976; Harrison et al., 2007). Children's ages ranged from 8 months to 8 years old (*M* = 4.3 years old).

Participant demographics were obtained prior to interviews (see Table 1) and used to guide recruitment following identification of the first participant in line with theoretical sampling used in GT (Strauss & Corbin, 1998). The initial phases used the demographics to seek as much variation as possible in the first few participants, followed by sampling based on concepts and themes emerging from the data in subsequent phases. As an example of the latter; as employment began to emerge as an important variable for adjustment, the next participant was specifically selected as a father who was in full‐time employment to explore this in more detail with them. Further parameters included birth order and age of the child as these appeared to initially impact upon fathers’ adjustment.

**Table 1:** Participant demographics

No.	Age	Age of child	Number of children	Birth order of child with DS	Type of employment
1	32	<1	1	First	Unemployed
2	46	4	3	Second	Full‐time
3	36	5	2	First	Full‐time
4	52	6	2	Last	Full‐time
5	47	4	3	First	Full‐time
6	41	5	3	Second	Full‐time
7	43	2	2	Second	Unemployed
8	40	5	2	First	Full‐time
9	46	3	3	Third	Unemployed
10	40	8	2	First	Part‐time
11	38	2	2	Second	Full‐time
12	26	2	1	First	Full‐time
13	45	7	2	Second	Full‐time
14	42	5	4	Third	Full‐time
15	35	6	2	First	Unemployed

After the first interview and throughout recruitment, participants with different demographics (e.g., age, number of children, employment status) were selected in order to add variation to the data, and to allow the exploration of emerging analytical themes.

## 8 RECRUITMENT

Prior to ethical approval being granted, the lead researcher liaised with group leaders from DS support groups to assess the feasibility of the study. Post ethical approval, fathers attending support groups were given copies of the participant information sheet and those interested were contacted and given a consent form at least 24 hr prior to each interview. Participants’ demographics were taken, to guide recruitment, and any queries about the study were addressed by the lead researcher. A consent form was reviewed and signed by the participant and lead researcher at the time of interview.

## 9 PROCEDURE

### 9.1 Ethical considerations

Ethical approval was granted by the University of Liverpool's Doctorate in Clinical Psychology Research Committee.

Throughout the study, participants’ confidentiality was maintained. All interviews were digitally recorded by the lead researcher and transcribed verbatim. Participant names were omitted during recording to preserve anonymity, and participants were allocated a unique identifier. Other names that were potentially identifiable were replaced with pseudonym post transcription.

It was agreed that, should participants become distressed during the interview, they would be signposted to appropriate support services. Participants were aware of their right to withdraw at any time and no participant highlighted distress during interviews.

### 9.2 Research interviews

Each participant chose the location of the interview, usually at their home, which lasted on average 1 hr. Interview duration gradually reduced throughout data collection, as expected with the methodology (Polit & Beck, 2003), and the shortest interview lasted 40 min. Semi‐structured interviews were guided by an initial schedule which provided a broad, flexible approach to exploring fathers’ experiences. The initial interview schedule was discussed with a second reviewer and piloted with a father of a child with DS who did not fulfil the geographical inclusion criterion for the main study. After each interview, the lead researcher noted hypotheses, reflections, a general summary and critique and learning points for future interviews. Participants’ narratives were transcribed and coded prior to the next interview to allow exploration of concepts in subsequent interviews.

The initial semi‐structured interview schedule broadly explored paternal parenting experiences and their adjustment to parenting a child with DS based loosely on the research questions (e.g., “What are the good things about being a dad to [child's name]?”;“Tell me a little bit about your caring responsibilities at home”; “How do you and the family cope? What helps/does not help?”; “How did you react when your child with DS was born? Where there any particular concerns you had?”; “What were your hopes/fears and how have these changed?”). The schedule was refined after the fifth interview and a second schedule developed to allow a more directive focus with subsequent interviews, and to stimulate theory development (Glaser & Strauss, 1999). This new schedule enabled a more direct focus on some of the emerging themes. The second interview schedule therefore included more directive questions, such as “One of the aspects of adjustment that previous participants have talked about is making adjustments/accommodating to their child. Thinking about your journey from birth to now, does that make sense to you?” “If so, what does accommodating to your child look like?” and “What does that concept mean to you?”. Participants were asked further questions such as “What helps you accommodate to your child? Is there anything which makes it easier?” and “What does not help? Is there anything that makes it difficult?”.

The authors agreed that theoretical saturation (Glaser & Strauss, 1999; Strauss & Corbin, 1998) of the major categories was reached by interview 10 within this study due to no new data emerging. The further five interviews were therefore used to refine and develop the theory.

## 10 ANALYSIS

The analysis of interviews was supported by use of NVivo 10 (QSR International, 2012), to aid identification and collation of codes and themes.

In accordance with the GT approach, data collection and analysis occurred simultaneously (Glaser, 1992; Strauss, 1987) following Strauss and Corbin's (1998) three stages of analysis outlined earlier: open coding; followed by axial coding; and finally, selective coding. The coding system was developed individually by the first author and through further discussion with the second author in supervision. Constant comparison, comparing new data with data already collected and coded, to identify emerging patterns, themes and concepts (Glaser, 1992), is an integral part of GT and occurred throughout. Consistent with Strauss and Corbin's (1998) approach, the analysis also utilized memos, and modelling of key themes, to aid later theory development.

During open coding, the first five interviews were subjected to micro analysis, which allowed the curiosity of the researcher to develop and provide the initial analytic direction, defining what was happening in the data. Segments of data were named that categorized, summarized and accounted for each piece of data for example “preparing for the future” to facilitate the identification of implicit concerns and explicit statements within the data. The majority of the codes constructed from the data used active language to capture processes and in vivo codes using participants’ own language. Reflection on interview memos enabled the clustering of codes into larger categories and subcategories. A storyline was created to capture the first five narratives and contribute to a composite narrative and initial, tentative model.

Axial coding followed, whereby relationships between each of the categories and subcategories were explored, allowing for the construction and testing of relational hypotheses over the next five interviews. The author sorted, synthesized and organized large amounts of data for example participant statements in an attempt to re‐assemble the data into new coherent components. Due to the minimal changes that occurred within the initial model, the final five interviews continued with the focus on relational hypotheses, exploring the existence of a category hierarchy, and identifying which categories appeared most important for adjustment.

Selective coding enabled the testing of relationships between the major categories and the overall model. However, no new information was uncovered with respect to understanding fathers’ experiences.

**Figure 1:**
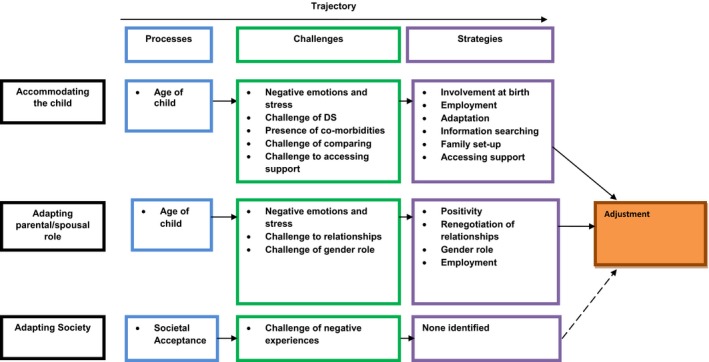
Theoretical model of fathers’ adjustment to parenting a child with Down's syndrome) [Colour figure can be viewed at http://wileyonlinelibrary.com]

Model development occurred in parallel with developments in the coding structure. The initial theoretical model of participants’ experiences, which was produced after the first five interviews, informed the modification of the interview schedule for the following five interviews. A second model was then produced which provided the focus for the final five interviews. Salient emerging data were incorporated into the second model, and a theoretical account of all participants’ experience was developed (Figure 1).

## 11 RESULTS

A narrative summary of the theoretical storyline is provided below, describing how fathers adjust to having a child with DS. The major conceptual categories, and subcategories, which are conceptualized as properties and dimensions (D) in line with Strauss and Corbin's (1998) approach are also outlined below. Quotations (presented in italic font) provide support for the author's interpretations and words within “[]” have been added to provide clarity, while “…” indicates the omission of text to provide a quotation appropriate in length.

## 12 NARRATIVE SUMMARY OF THE MODEL

Participants all reported they had made a positive adjustment to parenting their child with DS. Analysis of participants’ narratives led to the identification that parenting a child with DS is on a fluid trajectory, highlighting that adjustment has a course that changes over time for each father. Being a father to a child with DS presents challenges that can hinder the adjustment process, and fathers have employed deliberate strategies to shape their adjustment course and ultimately achieve positive adjustment.

There are three categories which feed into this trajectory: “Accommodating the child”; “Adapting the parental/spousal role”; and “Adapting society.”.

## 13 ACCOMMODATING THE CHILD

The first category that all fathers felt was fundamental to their overall adjustment was “Accommodating the child,” which is influenced by their child's age and begins at birth.

Fathers identified several challenges which dictated how demanding this process could be, and how successful accommodation is achieved. “Negative emotions and stress” conceptualized the “emotional rollercoaster” of parenting a child with DS, with especially difficult emotions being present at birth which faded as their child grew older. Fathers also described the challenges related to their child having DS and in particular how the “Presence of comorbidities” impacted on their ability to accommodate. Other challenges included the “Challenge of comparing” their child to others and the “Challenges to accessing support.”.

In light of the above challenges, fathers spoke about strategies which enabled them to accommodate, such as “Involvement at birth” which facilitated the development of a father‐child bond. The presence of siblings and birth order of the child with DS was also considered important (“Family setup”). Fathers also referenced how “Employment” had impacted on their ability to accommodate to their child, highlighting the importance of employer support and flexible working arrangements. Additional strategies included “Adaptation” (i.e., practical and emotional changes), “Information searching” and finally “Accessing support” all of which captured the positive steps fathers make to overcome the challenges encountered.

## 14 ADAPTING THE PARENTAL/SPOUSAL ROLE

A second category feeding into adjustment was “Adapting the parental/spousal role” which included the adaptations fathers make as a parent and how they actively negotiate and renegotiate their spousal relationship (“Renegotiation of relationships”).

In a similar way to “Accommodating the child,” this category was influenced by the “Age of the child” and the challenge of “Negative emotions and stress.” Additional challenges included “Challenges to relationships” and “Challenges of gender role,” whereby fathers described the impact of having a child with DS on their relationships, and how being male impacted upon the adaptations made.

To facilitate their adaptations, fathers employed a number of strategies including recognizing the positives (“Positivity”) and employed strategies related to their “Gender role” and their “Employment.”.

When exploring the temporal relationship between these two categories, most fathers identified that “Accommodating the child” and “Adapting the parental/spousal role” were equally as important, with no consensus as to the timing of each.

## 15 ADAPTING SOCIETY

A third category, “Adapting society” was included in the model for completeness, although the majority of fathers did not find this fundamental in terms of their adjustment process. Nevertheless, all fathers emphasized how increased societal awareness and acceptance of DS positively influenced on their adjustment.

Fathers’ experiences of adjusting to parenting a child with DS are depicted in Figure 1.

## 16 ACCOMMODATING THE CHILD

The process of “Accommodating the child” was influenced by the “Age of the child” (D). Participants described a number of challenges that impacted on their ability to accommodate to their child. These included: experiencing “Negative emotions and stress”; “Challenge of DS”; “Presence of co‐morbidities” (D); “Challenge of comparing” and; “Challenges to accessing support.” To overcome these challenges fathers utilized strategies such as: “Involvement at birth”; “Employment” (D); “Adaptation”; “Information searching”; “Family setup” and “Accessing support.”.

### 16.1 Age of child

The child's age seems to dictate fathers’ position on the trajectory and the accommodations made.

When he was younger…an issue was the medical side. As they get older…you’re more focussed on behaviour and how well they’re doing… so it’s changed over time (Participant [P] 6, 83‐8)

### 16.2 Negative emotions and stress

The challenge of negative emotions and stress that come with having a child with DS were emphasized with one participant describing their experiences as an “emotional rollercoaster” (P5, 412).

Fathers reflected on their feelings when learning of the DS diagnosis, with one participant describing it as a “grieving process…you feel it's like the end of the world” (P7, 267–71) and another describing difficulties accepting the loss of “a perfect child” (P14, 199–200).

Feelings of helplessness and uncertainty contributed to fathers’ stress:

No matter what you say having DS does have an influence…you think…what’ll happen when I’m not here (P2, 201‐4)

### 16.3 The challenge of Down's syndrome

One aspect contributing to fathers’ stress was the challenges related to their child's developmental delay associated with DS (e.g., communication, toilet training). One participant mentioned the more child‐related challenges, the “more interventions” (P8, 63) are needed.

### 16.4 Presence of co‐morbidities

Another challenge for fathers is the presence of the child's co‐morbidities which seem to slow down the process of “Accommodating the child,” (i.e., the more physical health complaints the child has, the harder it is to accommodate).

Within this property, an inherent strategy described by fathers was the use of downwards comparison whereby they described feeling “lucky” (P8, 72; P10, 198; P13, 44) when their child did not present with additional health problems. One participant explicitly noted the lack of co morbidities for his child.

helped [him] come to terms with things much quicker (P11, 14)

### 16.5 Challenge of comparing

Although downwards comparison is highlighted as a positive strategy above, most fathers described the upward comparisons they made between their child and other children without DS:

It doesn’t help in that your comparing [him] all the time to what they can do…then you’re more upset…that you’ve let yourself get upset (P4, 381‐6)

### 16.6 Challenges to accessing support

Participants identified that accessing professional and family support can be challenging; contributing to stress and impacting on their ability to accommodate their child.

A number of participants expressed feeling let down by professional support and described a battle for services, in light of budget cuts and an apparent “postcode lottery”.

A team of specialists should be helping her…so if the GP doesn’t come up with anything I’ll have to go and see the MP (P7, 235‐7)

Most participants felt they could challenge services if they were struggling, although one recognized that

not everyone is capable of being a strong advocate for their child (P9, 310‐11)

Further, the limited services available were perceived to disregard fathers in terms of their location, focus or the timing of support, which were all more mother orientated.

Going to some of the groups…the response I’d get from parents…was always that you know you’re a bloke and there was a bit of wariness…parents sort of keeping their child away (P10, 332‐7)

Fathers adopted a number of strategies related to these challenges which will now be presented.

### 16.7 Involvement at birth

Although most participants recalled their initial shock upon discovering their child had DS, they still mentioned they

couldn't imagine not being there (P2, 192‐3)

### 16.8 Employment

The challenges in accommodating their child appeared to be exacerbated by increased working hours. Flexible and supportive employers were credited as facilitating positive adjustment.

### 16.9 Adaptation

Another strategy that participants felt important was making practical (e.g., giving up hobbies) and emotional adaptations (e.g., embracing the role of becoming a father).

Most fathers recognized the close paternal bond they have with their child and the view that regardless of what disability their child has, they are still their child.

Another emotional adaptation is accepting that their child has DS which involves an adjustment of their expectations, a refocus on the positives and utilization of downwards comparison:

We get in the car and we kind of go phew glad I’m not X’s mum…we got off lightly. (P6, 325‐8)

A number of participants also believed their attitude and perspective on life was an important predictor of their adjustment, describing changes in attitude and becoming mindful since having a child with DS. Being mindful and taking “each day as it comes” (P1, 243–4) enabled accommodation to their child. One participant highlighted.

…you just cope, ‘cos the alternative is to just put your hands in the air and cry (P5, 420‐1)

### 16.10 Information searching

This strategy begins at the child's birth when fathers search online for information about DS:

the first thing you do is go on Google and understand what’s going on (P3, 445‐8)

This strategy seems to be relied upon less as the child grows older, replaced by seeking interpersonal support.

### 16.11 Family set up

For participants who had more than one child, the family setup and subsequent birth order of children was considered important.

Sibling competition is not a bad thing…he knows…Billy can do that I want to be able to do that (P9, 84‐92)

### 16.12 Accessing support

To overcome the challenges regarding accessing professional support outlined earlier, support was sought from elsewhere (i.e., from family and the “DS community”) (P4, 450). One participant described:

Going off meeting other parents… it’s quite an interesting disability because there’s a lot of us about (P5, 190‐2)

Most, but not all, participants referenced the benefits of attending groups to learn from other parents, with one participant referring to his group as “family” (P12, 91‐92).

## 17 ADAPTING PARENTAL/SPOUSAL ROLE

An important finding of this study was that in order for fathers to adjust, there are additional factors external to the father–child relationship considered important, such as relationship changes with their spouse and other children. As outlined in the previous domain, the “Age of child” impacts upon the timing of fathers’ need and ability to adapt to his role. Challenges that exist in relation to fathers adapting their roles include: “Negative emotion and stress”; “Challenges to relationships”; and the “Challenge of gender role.” Fathers appear to employ strategies to overcome these challenges such as: “Positivity” and the “Renegotiation of relationships.” Further, strategies are related to fathers’ “Gender role” and fathers’ “Employment” (D).

### 17.1 Age of child

Most participants recognized that their spousal relationship changes as their child develops. One participant stated:

It also helps…we've had a lot of time to adapt as a couple and actually the creases from the early days have been ironed out and we've changed…because we've overcome challenges together and in those early days talked through how we can make it work (P14, 153‐6)

### 17.2 Challenge of negative emotions and stress

Similar to the previous category, “Negative emotions and stress” impacted upon a participant's ability to adapt their roles. A few participants experienced guilt for not being as involved as their partner:

you can’t always be there…and you feel guilty (P2, 470‐1)

Some participants referred to the difficulties in talking to their partners, one participant summarized:

we bottle things up and maybe don’t communicate to each other as well as we could (P3, 210‐12)

### 17.3 Challenges to relationships

Participants outlined how:

personal relationships struggle, you don’t have much time for each other (P4 289‐292):

Just getting away for a night…you can’t leave Jay alone. In 6.5 years we’ve spent two nights away from Jay and that wasn’t far away (P4, 289‐292)

### 17.4 Challenges of gender role

The challenges that arise from being male were discussed. Most participants considered themselves the “breadwinner” (P2, 56), with mothers being the main caregivers, despite participants emphasizing the importance of equal roles. One participant explained that.

the stereotype of the female carer [still exists]…a lot of the dads step back (P10, 390‐3)

One participant stated:

Professionals…need to learn dad’s have just as much responsibility as mum…we’re fighting like hell to be accepted by the professionals but they’re just turning away because you’re not the mother (P12, 241‐6)

### 17.5 Positivity

It is important to note that despite the challenges participants identified in adjusting to parenting a child with DS, many positives were credited as facilitating their adaptation:

If I couldn’t notice all those positives about her as a mum and what we bring as parents then I wouldn’t be in the position I am today (P7, 401‐3)

### 17.6 Renegotiation of relationships

A further overall strategy concerned fathers’ relationship changes, with a few fathers highlighting how their child with DS had brought their spouse and themselves “closer together” (P1, 688).

A key change to relationships involved “Sharing caring responsibility” facilitated by setting routines and roles soon after their child's birth.

### 17.7 Gender role

In regards to fathers’ relationships with their spouses, and linked to the “Challenges of gender role,” participants spoke about staying strong to protect their partner:

I’d…[cry] outside so then she couldn’t see me (P1, 323‐5)

This could be seen as a, potentially maladaptive, strategy to help fathers’ adjustment by not burdening their spouse.

### 17.8 Employment

Fathers in full‐time employment explained how tiredness impacts on their ability to share caring responsibility:

I should and…could do more which makes me feel bad but I struggle with how busy my job is (P4, 56‐57)

Therefore, participants found it helpful to have a “reasonable employer” (P6, 549) who provided time off for appointments. A number of participants had changed jobs or working patterns, with three becoming full‐time primary carers for their children.

## 18 ADAPTING SOCIETY

As mentioned previously, only a minority of participants felt that “Adapting society” was fundamental to their overall adjustment.

### 18.1 The challenge of negative experiences

Negative experiences were mainly linked to the circumstances around their child's birth and the communication of the DS diagnosis:

the docs just didn't communicate it right…we had no clue what DS was (P12, 15‐18)

### 18.2 Societal acceptance

Conversely, participants described how societal views had changed for the better and how this helped counteract negative experiences:

I feel fortunate that we’ve had her now and not 20 years ago ‘cos it just seems a lot’s happened in… 20 years (P3, 488‐9)

## 19 DISCUSSION

The study aims were met through developing a model to account for fathers’ adjustment to parenting a child with DS. This is the first study to produce a theoretical framework outlining the processes of adjustment specifically for fathers of children with DS, outlined in the “Results” Section above. This model highlights the three important elements to achieving positive adjustment: accommodating the child, adjusting the parental/spousal relationship and adapting society. Within each of these discrete domains, these fathers encountered challenges which required them to employ specific strategies to overcome them. Given the limited evidence base specifically for DS, these findings will be situated within, and compared to, the broader existing evidence base of ID that characterizes adjustment as a global concept (Baker et al., 2005).

The overall analysis of participants’ narratives supports previous research that suggests adjustment to parenting a child with a disability is determined by the child's age and achieved over time (Carpenter & Towers, 2008; Lanfranchi & Vianello, 2012). This study has further added to the evidence base by highlighting that, for positive adjustment to occur, fathers have to adjust not only to their child (Gallimore et al., 1993), but also adjust their parental/spousal role. The links between fathers’ adjustment and their spousal relationship are supported by previous research conducted with fathers of children with DS (Cohen et al., 2016; Hornby, 1995).

Similar to findings from a study exploring fathers of children with ID (Lanfranchi & Vianello, 2012), “Adapting society” was identified as important, although most fathers of children with DS felt their child's acceptability was not pertinent to their overall adjustment. This was potentially as a consequence of the “DS advantage,” and the public's understanding of DS (Derrington et al., 2013; Hartley et al., 2012).

Across all three categories in the model, several challenges and strategies were captured. From the challenges described throughout the interviews, it was apparent that fathers *are* playing an active role in family life through providing care for their children and also supporting their spouse, in contrast to previous suggestions (Bailey et al., 1992). Despite their active involvement, however, fathers still feel secondary to mothers, suggesting very little has changed in 20–30 years (Lillie, 1993; Parette et al., 2010).

This study also highlights that fathers *do* experience stress, particularly in the early days, but this is generally hidden through using coping strategies such as concealing their emotions (Barak‐Levy & Atzaba‐Poria, 2013; Cummings, 1976; Houser & Seligman, 1991; Pelchat et al., 2003; Rodrigue et al., 1992).

The early days, in particular their child's birth and diagnosis were a particular challenge for participants who experienced uncertainty and grief, which has been highlighted in previous reports (Baxter et al., 1995; Gault, 2009; Herbert & Carpenter, 1994). The limited information provided at birth exacerbated feelings of helplessness, and fathers consequently relied on the Internet for support. As their children develop, fathers’ challenges become more associated with seeking professional support and balancing childcare demands with employment, matching previous findings (Baxter et al., 1995; Hastings, 2003).

Similar to the findings of Houser and Seligman (1991), but in contrast to Rodrigue et al., (1992), fathers did not reference the gender of their child as a challenge to their adjustment.

Despite experiencing challenges, fathers were able to actively manage and overcome these by using a range of strategies which enabled them to positively adjust with love and commitment towards their child. Consistent with previous studies, fathers spoke of personal growth, attributing this to their strong paternal bond and the positive changes they had made (Bentley et al., 2015; Carpenter & Towers, 2008; Hornby, 1992).

In contrast to Cohen et al.’s (2016) study which suggested that fathers’ stress remained static, fathers in the current study indicated otherwise. The differences in findings could be attributable to the different methodology used as Cohen et al.’s (2016) study utilized questionnaires. Fathers’ coping strategies also appeared to change over time, in support of Rendall's (1997) and West's (2000) conclusions. For example, most fathers found their reliance on the Internet was replaced by attendance at support groups. In support of previous research, fathers in this study focused pragmatically on challenges by just “getting on with it” and planning for the future (Bentley et al., 2015; Herbert & Carpenter, 1994). As mentioned earlier, fathers mostly dealt with challenges alone, which links closely with research highlighting that when males experience distress, beliefs such as “I must not be weak” govern their behaviours (Endler & Parker, 1994; Kingerlee, 2012).

Although involvement at birth was not an active strategy fathers might have undertaken to facilitate adjustment, the successful outcomes fathers experience as a result should be communicated (Brown et al., 2012; Palm & Fagan, 2008; Pleck & Masciadrelli, 2004). Similarly, “Employment” might not have been seen as an active strategy to facilitate adjustment, but participants highlighted how flexible and supportive employers impacted upon their adjustment trajectory, supporting previous findings (Hornby, 1995).

Another strategy fathers used to overcome challenges was renegotiating roles and relationships, involving sharing the caring responsibility; a finding that is supported by previous research (FLPD, 2005; West, 2000).

In summary, this study supported previous findings that, despite the emotional and practical challenges that come with parenting a child with DS, fathers can and do positively adjust (Bentley et al., 2015; Henn & Piccinini, 2010). This paper further adds to the evidence base by being the first study to propose a broad theoretical model for fathers’ adjustment to parenting a child with DS, highlighting the challenges involved to achieving positive adjustment and the strategies fathers employ to overcome these.

## 20 CLINICAL IMPLICATIONS

Based on the experiences of current participants, there remains a gap in service provision which suggests more could be done to involve and support fathers. This study supports the increasing trend of paternal involvement in the care of their disabled child, which needs to be acknowledged by services, and parental couples approached as equal partners.

It is important for professionals to recognize positive parenting experiences and equally support the minority of fathers who do require specialist intervention (Hornby, 1995; Houser & Seligman, 1991). If fathers do access support concerning their adjustment, services could acknowledge the two, potentially three, key domains that have been elucidated here. This supports previous research by Bentley et al. (2015) who highlighted how clinicians can support fathers to experience growth and self‐development by taking a non‐deficit approach to their experiences. It might be true that the most appropriate intervention which could be offered to fathers experiencing challenges might differ, depending on individual characteristics and circumstances. However, there may be some benefit to offering this to fathers within a group setting, where others experience similar challenges and difficulties.

Supporting previous research, this study has identified the benefits of non‐professional support and thus services should consider how best to signpost fathers to support groups and empower them to access support, particularly within their own family (Cohen et al., 2016; Gault, 2009; Herbert & Carpenter, 1994; Hornby, 1995). Spousal support and satisfaction evidently impacts upon fathers’ experiences and in support of recommendations made by previous researchers, services should seek to help both parents to facilitate supportive relationships (Cohen et al., 2016; Hornby, 1995; Rodrigue et al., 1992).

A recommendation specifically for medical professionals, particularly at the child's birth; is to involve fathers and communicate the implications of a DS diagnosis by stating facts and focusing on the *positive* attributes, which in the past have been neglected by research, that having a child with DS can bring. For example, fathers referenced personal growth, their strong bonds with their children and how having a child with DS has strengthened their relationships. Participants felt this would have reduced their anxiety and limited their risk of finding inaccurate, and even potentially detrimental information, online (Gault, 2009).

Additionally, participants talked about the importance of being present at their child's birth to strengthen future bonds and help accelerate the adjustment trajectory. It is therefore important that midwives communicate this to fathers who have become aware that their child has DS through prenatal testing.

## 21 STRENGTHS AND LIMITATIONS

This is the first study within the United Kingdom which offers an understanding of fathers’ adjustment to parenting a child with DS, highlighting the interest and motivation of fathers to participate in research. Importantly, this study has addressed a major criticism identified with many studies in this area (e.g., the use of mothers to represent “parent views”).

The study benefited from GT methodology with a strength including the use of theoretical sampling. This allowed the in‐depth exploration of fathers with different experiences and backgrounds, highlighting how individual‐level factors, such as psychological resources (e.g., downwards comparison) and community resources (e.g., flexible employment) work to influence fathers’ experiences. Conversely, the use of qualitative research methods, and in particular theoretical sampling, may limit the generalizing of findings to other populations.

Another limitation which should be recognized is the self‐selecting sample consisting of mainly White British fathers in financially stable jobs. It could be hypothesized that fathers attending support groups and volunteering to participate in research may be more likely to feel able to communicate their experiences, or hold particular views. Their employment status may also enable them to better support their family financially. It could also be hypothesized that fathers who refused to take part may have had different experiences, or not achieved positive adjustment. In common with all research, it is also important to consider the possibility of “social desirability” in the fathers’ responses to questions about their family life, coping and challenges.

## 22 FUTURE RESEARCH

Future researchers need to utilize the strong desire of fathers to participate in research, evident in this study. There is a need to explore fathers’ experiences across varying diagnostic categories, rather than grouping fathers under one umbrella of ID, where specific implications might be lost (Cuskelly, 1999).

Additionally, there needs to be consideration about how researchers can reach fathers not obtaining support to capture their adjustment experience, especially those who might be struggling. Fathers who are not currently residing with their partners and child are of particular interest, potentially providing insight into adjustment challenges and what may have led them to leave the family home. Recruiting from other geographical areas and cultures may provide further variations in the findings.

Although there are numerous studies exploring mothers’ experiences of parenting a child with an ID, it would be interesting to explore mothers’ adjustment specifically to a child with DS, to see whether similarities exist with the adjustment domains, and to explore the complexities of parental relationships from their perspective.

Further replication studies would add strength to the findings of this study and are warranted to explore why adapting society is important for some fathers but not all.

## 23 CONCLUSION

In conclusion, this study contributes to a neglected literature base, through providing the only GT study to explore the adjustment trajectory for a previously hidden group of fathers who, despite experiencing challenges, have positively adjusted, demonstrating strength, resilience and commitment to their children and families.

It is hoped this study will help to raise awareness of the importance of the fathers’ roles when parenting children with DS, whilst highlighting the need for further father focused research.
